# Information and communication technology-based health interventions for transgender people: A scoping review

**DOI:** 10.1371/journal.pgph.0001054

**Published:** 2022-09-15

**Authors:** Horas T. H. Wong, Sujith Kumar Prankumar, Jialiang Cui, Christopher Tumwine, Isaac Yeboah Addo, Wansang Kan, Muhammad Naveed Noor

**Affiliations:** 1 Susan Wakil School of Nursing and Midwifery, The University of Sydney, Sydney, Australia; 2 The Albion Centre and t150 Transgender Health Service, NSW Health, Surry Hills, Australia; 3 Centre for Social Research in Health, UNSW Sydney, Kensington, Australia; 4 ARC Centre of Excellence for Automated Decision-Making and Society, Swinburne University, Melbourne, Australia; 5 Department of Social Work, Chinese University of Hong Kong, Hong Kong, Hong Kong; 6 Department of Mental Health, School of Medicine, Kabale University, Kabale, Uganda; 7 The Cancer Institute NSW, Sydney, Australia; 8 Department of Pathology and Laboratory Medicine, Aga Khan University, Karachi, Pakistan; Anglia Ruskin University - Cambridge Campus, UNITED KINGDOM

## Abstract

In the recent past, there has been a strong interest in the use of information and communication technology (ICT) to deliver healthcare to ‘hard-to-reach’ populations. This scoping review aims to explore the types of ICT-based health interventions for transgender people, and the concerns on using these interventions and ways to address these concerns. Guided by the scoping review frameworks offered by Arksey & O’Malley and the PRISMA-ScR checklist, literature search was conducted in May 2021 and January 2022 in three databases (PubMed, CINAHL and Scopus). The two searches yielded a total of 889 non-duplicated articles, with 47 of them meeting the inclusion criteria. The 47 articles described 39 unique health projects/programs, covering 8 types of ICT-based interventions: videoconferencing, smartphone applications, messaging, e-coaching, self-learning platforms, telephone, social media, and e-consultation platforms. Over 80% of the health projects identified were conducted in North America, and 62% focused on HIV/sexual health. The findings of this review suggest that transgender people had often been regarded as a small subsample in ICT-based health projects that target other population groups (such as ‘men who have sex with men’ or ‘sexual minority’). Many projects did not indicate whether transgender people were included in the development or evaluation of the project. Relatively little is known about the implementation of ICT-based trans health interventions outside the context of HIV/sexual health, in resource limiting settings, and among transgender people of Asian, Indigenous or other non-White/Black/Hispanic backgrounds. While the range of interventions identified demonstrate the huge potentials of ICT to improve healthcare access for transgender people, the current body of literature is still far from adequate for making comprehensive recommendations on the best practice of ICT-based interventions for transgender people. Future ICT-based interventions need to be more inclusive and specified, in order to ensure the interventions are safe, accessible and effective for transgender people.

## Introduction

In recent years, there has been strong interest in the use of telemedicine to deliver healthcare. Telemedicine (sometimes referred to as ‘telehealth’ or ‘e-health’ [1]), in its broadest sense, refers to the use of information and communication technologies (ICTs) in the delivery of health services at a distance [2]. The use of telemedicine has surged during the COVID-19 pandemic due to the restriction of in-person interactions. Clinicians around the world have used different ICT-based platforms, including computers, mobile phones, and the Internet to address some of the challenges faced by developing and developed countries in providing high-quality, accessible and cost-effective health care services [3–5].

One of the most notable advantages of telemedicine is increasing access to health care services for geographically dispersed, disadvantaged and stigmatized populations [6] such as transgender people [7, 8]. Transgender is generally defined as an ‘umbrella term that describes persons whose gender identity, gender expression or behaviors does not conform to that typically associated with the sex to which they were assigned at birth’ [9]. The transgender population is incredibly diverse, and the definition of transgender is culturally specific [10]. Transgender people may include individuals who undergo gender affirmation surgery and/or receive gender-related medical interventions like hormone therapy, and who identify as having no gender, more than one gender or alternative genders [11]. For these reasons, it is difficult to estimate the actual population of transgender people. Some previous epidemiological and clinic-based studies have estimated that between 0.1 and 2% of the population identified as being transgender or other forms of non-cisgender identities [12–14].

It is well documented in the literature that due to intersecting forms of social marginalization and legal exclusion, transgender people are disproportionally affected by a wide range of human rights violations and adverse health outcomes [15]. Transgender people, especially those who are from a minority ethnic group, are disproportionately affected by gender-based hate crimes [16]. Minority stress research has shown that stressful events experienced by transgender people have made them more vulnerable to mental health problems such as depression, anxiety and suicidal ideation [17–19]. A lack of support systems in the society for transgender people can also contribute to their higher risk for substance misuses, HIV and other sexually transmissible infections (STIs) [17, 20–22].

Despite the increasing social acceptance of sexual and gender diversity in various parts of the world, transgender people often experience barriers to accessing adequate healthcare [23–26]. Some major barriers that impede transgender people’s access to healthcare include discrimination in healthcare settings, a shortage of gender care specialists, a lack of adequate health insurance, poverty, and societal stigma [27–30]. Telemedicine can address some of these barriers, for instance, by offering transgender persons flexible, safe, private and comfortable ways to connect directly with transgender health specialists virtually, thereby reducing the risk of discrimination experienced by transgender persons in the process of seeking healthcare [8].

There is an incipient body of literature that advocates for the use of telemedicine to improve transgender people’s health outcomes. Studies have shown an increased acceptance of telemedicine among transgender people and clinicians [31, 32], and suggest that telemedicine can help promote HIV and sexually transmissible infection (STI) testing and care and improve psychosocial wellbeing, by simplifying access to gender-affirming hormone therapy [7, 33, 34] and perioperative care to individuals who undergo gender affirmative surgeries [35]. Some recent literature reviews on the use of digital and mobile health interventions have suggested that such interventions can effectively promote transgender youths’ health [36, 37], increase HIV testing rates [38], and deliver gender-affirming care [34]. However, these reviews either focused on younger transgender people or a particular health issue (i.e., HIV testing or gender-affirming care). Our article aims to extend the existing literature by exploring the types of ICT-based health projects/programs and interventions that have been used to deliver health services to transgender people of different ages and the concerns on using these interventions and ways to address these concerns.

## Methods

### Protocol and research questions

The review was guided by the frameworks developed by Arksey & O’Malley [39] and the PRISMA-ScR checklist [40]. The full protocol of this scoping review has been published elsewhere [41]. The overarching research question that guided this review was: *What does the extant research say about the delivery and receipt of transgender health services through digital means*? In particular, the following questions were explored:

1. What ICT-based health projects/programs have been documented in the literature and how have these projects/programs been used to deliver healthcare for transgender people?
2. What is lacking in the current body of literature on ICT-based health projects/programs for transgender people?
3. What are the concerns about effective ICT-based health projects/programs for transgender people and ways to address these concerns?

### Search strategy

We initially searched PubMed, CINAHL and Scopus for articles published in English, with additional grey literature explored using Google Scholar and citation mining, in May 2021. Our multilingual team also performed a trial search in the Chinese-language e-journal database, Airti Library, in the early stage of the review which returned no relevant publications, so we decided not to include the database. Search terms were developed iteratively through team meetings and trial searches and were grounded on three concepts: telemedicine (e.g., m-health, e-health, digital health, telehealth), transgender persons (e.g., transgender, gender diverse, transman, transfeminine, gender non-binary), and delivery of health care (e.g., health service delivery, health promotion, consultation, and access to care). A supplementary search was conducted in January 2022 to identify more recent articles published between May and December 2021. The complete search strategy is presented in S1 File.

### Inclusion criteria

Publications were only included in the review if they: 1) contained a detailed description of the development, implementation or evaluation of a particular ICT-based health project/program (i.e., services, interventions or research) that used digital means to improve the delivery of healthcare for transgender individuals (in other words, opinion pieces that only discussed the pros and cons of ICT use in a general sense, articles using ICT only as a means to recruit transgender participants, and research that studied the general attitudes of using ICT among transgender participants without describing a particular ICT-based project/program were not included); 2) successfully recruited or aimed to recruit transgender people, or if transgender people were direct recipients of the service/intervention/research project (i.e., articles discussing skills development for healthcare workers were not included); 3) were written in English.

### Selection of articles and data charting process

Duplicates were removed following the search. Article screening, reviewing and data extraction tasks were shared by all members of the team. First, titles and abstracts of the non-duplicated articles were shared equally to screen out papers that did not meet the inclusion criteria. The title and abstract of each article were reviewed by two members independently to minimise misclassification. Then, the full text of the shortlisted articles was shared equally between team members and was reviewed rigorously to screen out any remaining papers that should be excluded. Data extraction was performed alongside full-text article review and was guided by a pre-developed framework which consisted of publication information (authors, article type, country and year of publication), types of ICT intervention used, target audience, total number of participants, total number of transgender participants, enablers and challenges described, recommendations, limitations of study methodology, and key takeaways of the study. During the review and data extraction process, any doubts that arose in regard to the process were discussed in team meetings.

## Results

### Overview of included articles

**Fig 1:**
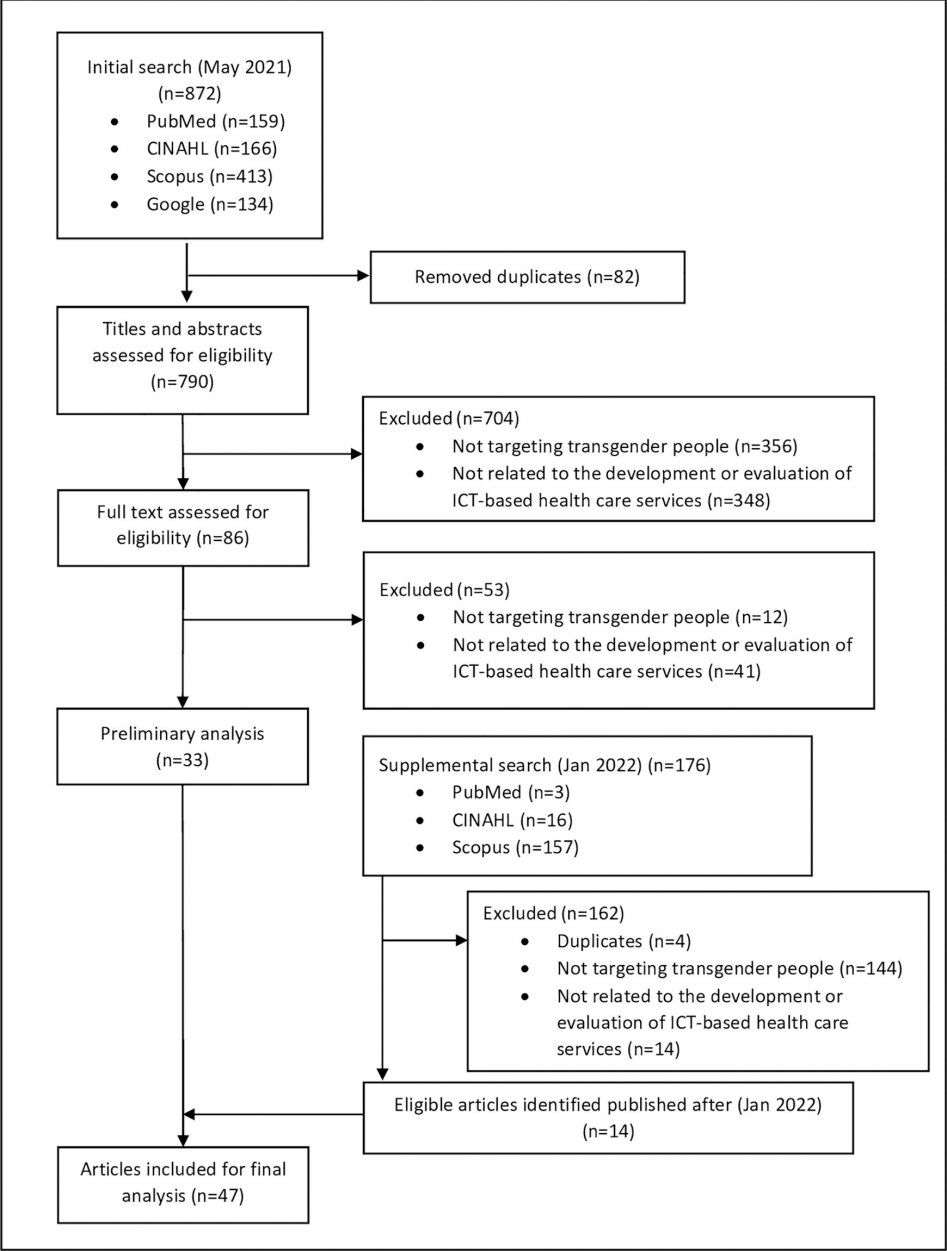
Flowchart of the review.

The initial search yielded 790 non-duplicate articles. After reviewing the titles and abstracts, 704 articles were excluded, leaving 86 for full text review. At this stage, 33 articles were included in the preliminary analysis. The supplementary search in January 2022 yielded additional 176 articles. After a further review, a total of 14 additional articles met the inclusion criteria and were included in the final analysis (i.e., final sample size = 47 articles) (Fig 1). All articles were published between 2014 and 2021, but more than half of these papers (n = 26) were published in or after 2020.

The 47 articles covered a total of 39 ICT-based projects, of which the characteristics are summarised in Table 1. Majority of these projects 34 (87%) were conducted in North America (USA, n = 32; Canada, n = 2), while three projects were conducted in Asia (Thailand, n = 1; Thailand + India, n = 1; and Indonesia, n = 1), and one in Australia. Over half (59%) of the projects had an experimental design (RCT, n = 15; quasi-experimental, n = 8). The earliest project identified was conducted in 2010 [80] but most (82%) began participant recruitment in or after 2015. Among all projects, 24 (62%) focused on improving HIV and/or sexual health, and 9 (23%) focused on gender-affirming care.

**Table 1:** Characteristics of the 39 projects identified from the articles.

Characteristics	N	(%)
**Country**		
USA	32	(82)
Canada	2	(5)
Thailand	2	(5)
Australia	1	(3)
India	1	(3)
Indonesia	1	(3)
**Project type**		
RCT	15	(38)
Retrospective data review	11	(28)
Quasi-experimental study	8	(21)
Qualitative evaluation	5	(13)
**Project commencement year**		
2020 or after	9	(23)
2015–2019	23	(59)
2014 or before	6	(15)
Unknown	1	(3)
**Main health dimension(s) focused on**		
HIV/Sexual health	24	(62)
Gender affirming care	9	(23)
Behavioural/psychosocial wellbeing	7	(18)
COVID health	1	(3)
**Targeted participants**		
TG only	15	(38)
Mainly MSM	15	(38)
LGBTIQ/Sexual minority	5	(13)
Others	4	(10)
**Other intersectional risks focused on**		
People living with HIV	6	(15)
People who use substance/drugs	2	(5)
Incarcerated or post-incarcerated people	2	(5)
People experiencing homelessness	1	(3)
**Transgender participant age or target age** ^**1**^		
Including 18 or below	5	(13)
Adults only	8	(21)
Unknown/ no specific target	26	(67)
**Most reported ethnicities or target ethnicities** ^**1**^		
Black/Latino/Hispanic	12	(31)
White	10	(26)
Asian	3	(8)
Others non-white minorities	2	(5)
Unknown/ no specific target	12	(31)
**Community consultation at the development stage**		
Transgender people were involved	9	(23)
Transgender people may be involved^**2**^	3	(8)
**ICT-based interventions involved**		
Videoconferencing	13	(33)
Smartphone apps	11	(28)
Messaging	9	(23)
e-Coaching	7	(18)
Self-learning platforms	6	(15)
Telephone call	3	(8)
Social media	3	(8)
e-Consultation platforms	2	(5)

^1^ ‘Target age’ and ‘target ethnicities’ are for ongoing projects that recruitment was underway.

^2^ For example, projects indicating the involvement from ‘key populations’, ‘sexual minority’, or an advisory board without specifying its composition.

### Characteristics of participants in the literature

Among the 39 ICT-based projects, 15 (38%) specifically targeted transgender people. There was one project which referred to ‘transfemale and gender-nonconforming people’ as ‘MSM’ (men who have sex with men) [42], and another project [43] which categorised ‘transgender’ (a gender identity) as a ‘sexual identity’ that was regarded as mutually exclusive with respect to being ‘gay’ and ‘bisexual’ (c.f. transgender people can be of different sexual orientations [10]). Some projects, despite stating that transgender people were one of their target audiences, only recruited a small number of transgender people or did not specify how many transgender people were reached or targeted. Among the projects that reported the transgender sample size, a total of 5927 (n = 1 to n = 1828) transgender people were recruited, and among them, 4338 (n = 0 to n = 1828) received an ICT intervention (e.g., they were assigned to the intervention arm of an experimental study) (Table 2).

**Table 2:** Overview of the 39 ICT-based trans health projects identified from the 47 articles (N/A = Not indicated in the article).

Project background							Participants						Interventions used	Key messages/Outcomes	Ref
Participant recruitment period	Type	Name	Country	Project aim	Main dimension	Target	Race/ethnicity	Sample size	Age of all participants	No. of trans participants	Age of trans participants	Trans community consultation			
2021-ongoing	RCT (Protocol)	#SafeHandsSafeHearts	Thailand, India	To increase COVID-19 knowledge & protective behaviours, & reduce pandemic stress	COVID health	LGBT+ people	Target: Thai, Hindi/ Marathi/ English speakers	Target: 618	Target:18+	Target 206	*N/A*	*N/A*	Videoconferencing e-coaching	(Project is ongoing)	[45]
2021	Retrospective data review	*N/A*	USA	Providing behavioural health care, therapy & support	Behavioural/psychosocial wellbeing	Clients visiting a LGBTQ+ organisation	*N/A*	*N/A*	*N/A*	*N/A*	*N/A*	*N/A*	Videoconferencing	Telehealth is an important, flexible & effective tool to deliver behavioural healthcare that can engage geographically diverse communities. There are some concerns regarding familiarity with technology, confidentiality & safety. The suboptimal interpersonal contact may be challenging for some staff & clients.	[46]
2020-ongoing	RCT (Protocol)	HealthMpowerment2.0	USA	To promote user-generated content, social support to reduce intersectional stigma, & improve HIV-related outcomes. A substantial focus on mental health	HIV/Sexual health, Behavioural/psychosocial wellbeing	Black MSM, & TG women aged 15–29	*N/A*	Targe 1050	Target 15–29	Target 10% of sample	*N/A*	Yes (2 TGW)	Smartphone application	(Project is ongoing)	[47]
2020	Retrospective data review	*N/A*	USA	Providing sexual health clinic consultations, including HIV/STI treatment & care, gender affirming care	HIV/Sexual health, gender affirming care	Clients visiting a LGBTQ+ clinic	*N/A*	*N/A*	*N/A*	*N/A*	*N/A*	*N/A*	Videoconferencing	Telehealth is a highly acceptable, satisfactory way to provide HIV & LGBTQ+ care during the COVID-19 pandemic for both clients & providers.	[48]
2020	Retrospective data review	*N/A*	USA	Providing gender affirming care	Gender affirming care	Gender diverse youth visiting a telemedicine gender clinic	White (95%) Hispanic/Latinx (9%) Black (2%) Asian (4%)	57	13–17	57	13–17	*N/A*	Videoconferencing	Telemedicine is a highly acceptable, satisfactory way to provide gender affirming care during COVID-19. Clients tend to prefer in-person consultation in the initial visits. There are some concerns regarding privacy & hesitation regarding camera use.	[32]
2019-ongoing	RCT (Protocol)	mLab	USA	To improve both HIV testing rates & linkage to care	HIV/Sexual health	Young MSM & TG women	Target Black, Latinx	Target525	Target 18–29	*N/A*	*N/A*	*N/A*	Smartphone application	(Project is ongoing)	[49]
2019-ongoing	RCT (Protocol)	GeoPassort	USA	To support HIV, STI prevention by facilitating access to needed services.	HIV/Sexual health	Currently or recently incarcerated MSM & TG women aged 18–49 who have substance use disorders	*N/A*	Target300	Target18-49	*N/A*	*N/A*	*N/A*	Smartphone application e-Coaching	(Project is ongoing)	[50]
2020	Retrospective data review	*N/A*	Canada	Providing gender affirming care	Gender affirming care	TG youth visiting a paediatric gender clinic	*N/A*	*N/A*	Under 18	*N/A*	Under 18	*N/A*	Videoconferencing	Evaluation found the service was highly acceptable by trans youth & their caregivers. The service was considered safer than in-person visit & save cost & travel time.	[51]
2020	Retrospective data review	*N/A*	USA	To provide gender affirming care including mental health services, hormones & speech therapy	Gender affirming care	TG patients of a health clinic	White (73%) Multiracial (6%) Black (6%) Asian (5%)	*N/A*	12–60+	3189	12–60+	*N/A*	Videoconferencing	Telehealth was a possible alternative for face-to-face gender affirming care. Removing state licensure requirements for telehealth could facilitate access.	[44]
2020	Quasi-experimental	Trans Women Connect	USA	HIV prevention & sexual health promotion	HIV/Sexual health	TG women aged 21–28	*Phase 1* "79% people of color” *Phase 3* White (38%), Asian (31%), Black (19%), American Indian/Alaska Native (19%)	73	18–59	73	18–59	Yes (3 TGW of color)	Smartphone application	A user-centred mHealth intervention is an acceptable & effective tool to engage TG women & promote HIV & sexual health. Increases in self-efficacy in finding lesbian, gay, bisexual, TG, & queer (LGBTQ)–friendly services; intention to seek online social support; & PrEP knowledge were statistically significant.	[52]
2020	Qualitative evaluation	LifeSkills Mobile	USA	To reduce HIV risk through self-directed activities	HIV/Sexual health	TG women aged 21–28	"Racial/ ethnic minority" (88%)	8	21–28	8	21–28	Yes ("expert" & young TGW)	Smartphone application	The app adopted a user-centred design. The intervention was highly acceptable.	[53]
2019–2020	Retrospective data review	*N/A*	USA	Gender affirming care other TG health	Gender affirming care	TG people who visited family clinics	*N/A*	374 (telehealth visits) 1454 (in-person visits)	*N/A*	374 telehealth visits 1454 (in-person visits)	*N/A*	*N/A*	Videoconferencing	Telehealth can help increasing access & availability of gender-affirming care even during COVID-19.	[54]
2019–2020	Retrospective data review	USAF telehealth program	USA	To provide trans health-related care	Gender affirming care	TG members of the Air Force	*N/A*	20	19–42	20	19–42	*N/A*	Videoconferencing	Using telehealth to deliver trans health was well received by USAF TG members locating in different countries, especially for areas with a paucity of trans health services.	[55]
2019	Qualitative evaluation	TransLife	USA	To help preventing suicide by help users self-monitor their mood & providing relevant resources	Behavioural/psychosocial wellbeing	TG persons	White (44%) Hispanic/Latinx (31%) Black (12%) Asian/Pacific Islander (12%)	16	Mean age (33.4)	16	Mean age (33.4)	Yes (with TG clients of a community centre [unknown numbers])	Smartphone application	The intervention was acceptable among participants.	[56]
2018–2021	RCT (Protocol)	TechStep	USA	To reduce condom-less anal sex, increase condom self-efficacy, & promote HIV testing	HIV/Sexual health	TG people aged 15–24	*N/A*	Target250	Target15-24	Target 250	*N/A*	Yes (a trans youth advisory board [composition *N/A*])	Smartphone application Messaging e-coaching	(Project is ongoing)	[57]
2018–2020	RCT (Protocol)	SMART	USA	To reduce condom-less anal sex, increase condom self-efficacy, & promote HIV testing	HIV/Sexual health	Adolescent MSM aged 13–18 (assigned male at birth, identify as sexual minority, attracted to cisgender male)	English & Spanish speaking	Target: 1285	Target: 13–18	*N/A* target	*N/A*	*N/A*	Videoconferencing Learning platform	(Project is ongoing)	[58]
2018	RCT	Singularities	USA	To improve help seeking & coping among sexual & gender minority youth	Behavioural/psychosocial wellbeing	Sexual and gender minority youth	White (62%) Latinx (21%) Asian/Pacific Islander (4%) Black (3%)	240	14–18	113 "Gender minority"	*N/A*	Maybe (3 "sexual & gender minority youth")	Learning platform	Using an online game-based intervention was feasible & acceptable for sexual & gender minority youth. It may also be effective in reducing binge drinking frequency, marijuana use & cyberbullying.	[59, 60];
2018	RCT	QueerVibe	UK	To empower users & improve psychosocial & physical wellbeing	Behavioural/psychosocial wellbeing	Trans-masculine & nonbinary youth	White (96%) Mixed (2%) Asian (1%) Black (1%)	156	15–21	156	15–21	Yes (9 TG persons)	Learning platform	The intervention showed significant improvements in self-empowerment & psychological & physical wellbeing.	[61]
2017–2019	RCT (Protocol)	*N/A*	USA	Promoting HIV prevention practices including condom use, PrEP & PEP use, & so on.	HIV/Sexual health	Gay, bisexual, TG, homeless, & post incarcerated youth at high risk for HIV	Target: African/ Latino	Target1500	Target 12–24	*N/A*	*N/A*	Maybe (a youth advisory board [*N/A* composition])	Messaging Social media e-coaching	(Project is ongoing)	[62]
2017–2018	Qualitative evaluation	T5K HIV-Positive Result Delivery	USA	Delivery of HIV-positive results by phone after home-based HIV testing	HIV/Sexual health	Cisgender MSM, & TG men & women who were just diagnosed with HIV	*N/A*	132	16–49	*N/A*	*N/A*	*N/A*	Telephone	Phone delivery of positive HIV-results is acceptable, at-home testing with phone delivery has the potential to increase HIV testing access, especially to geographically isolated or medically underserved populations.	[63]
2017–2018	Retrospective data review	eConsult	Canada	To link primary care providers & TG care specialists	Gender affirming care	Primary health provider	*N/A*	*N/A*	*N/A*	*N/A*	*N/A*	*N/A*	e-consultation platform	The e-consult service could significantly improve access to care for TG patient by decreasing wait-time.	[64]
2017	Retrospective data review	*N/A*	USA	To link clinicians with TG specialists	Gender affirming care	Clinicians of Veterans Affairs	*N/A*	*N/A*	*N/A*	*N/A*		*N/A*	e-consultation platform	The TG program is generally useful for clinicians to seek support from TG health specialists.	[65]
2017–2018	RCT	Project Moxie	USA	Providing video-chat counselling after home-based HIV testing	HIV/Sexual health	TG youth	Non-Hispanic white (66%) non-Hispanic non-white (21%) Hispanic (13%)	126 (intervention) 75 (control)	15–24	126 (intervention) 75 (control)	15–24	*N/A*	Videoconferencing	TG youth have low levels of HIV & STI testing. Video-chat counselling received general high level of satisfaction from participants. No statistically significant differences between control & intervention arms regarding PrEP willingness.	[66–68]
2017	Quasi-experimental	RUMAH SELA	Indonesia	Providing information on HIV transmission, testing, & condom use. Users can ask questions & being responded within 12 hours.	HIV/Sexual health	MSM, TG women, people who use drugs	Indonesian (100%)	168	16–30	49	Mean age (26)	Maybe (20 members of "key population")	Smartphone application	A peer-customised mHealth app based on self-learning principles is a cost-effective intervention to increase HIV-related knowledge, condom use, & HIV testing. It may also improve self-esteem of TG people.	[69]
2017	Quasi-experimental	*N/A*	USA	To pilot test & develop a telehealth intervention targeting TG women of color for gender affirming care	Gender affirming care	TG people who were assigned male at birth	Black (96%) Hispanic/Latinx (4%)	25	18+	25	18+	Yes (13 TG persons)	Videoconferencing Messaging Telephone e-coaching	Telehealth access with a peer health consultant can significantly increase in the intention to seek transgender-specific care, HIV care & mental health care	[70, 71]
2016–2020	Retrospective data review	PrEPme Virtual outreach	USA	Increasing PrEP awareness & HIV prevention & to enrol PrEP intake.	HIV/Sexual health	LGBTQ+ people in Baltimore City	Black (58%) White (39%) Hispanic (8%) Asian (4%)	26	*N/A*	4	*N/A*	*N/A*	Smartphone application T	Online outreach may have greater success in enhancing people’s linkage to care compared to offline outreach	[72]
2016–2019	Quasi-experimental	HealtheNav	USA	To provide support to help newly diagnosed MSM & TW to navigate HIV care	HIV/Sexual health	Newly diagnosed HIV-positive MSM & TG women aged 18–34	Black (18%) Hispanic/Latinx (32%) White (27%)	120	18–34	17	*N/A*	*N/A*	Messaging e-coaching	The project was able to deliver personalised social support to and collect real-time data from participants. Some tips for implementation included being flexible and responsive, involving peers, and using direct and succinct messages.	[73]
2016–2018	RCT	Text me, Girl!	USA	To improve HIV-related outcome & virological suppression	HIV/Sexual health	HIV-positive TG women	Black (43%) Latinx (38%) Others (20%)	130	19–34	130	19–34	Yes (An advisory board of TGW of various backgrounds)	Messaging	A unidirectional, automated text-messaging intervention could improve HIV care outcomes among young adult transwomen living with HIVV, including improving retention to HIV care, viral suppression & ART adherence.	[74, 75]
2016–2017	Quasi-experimental	weCare	USA	Health educators use ICTs to better engage with racially/ethically young gay & bisexual men & TG women in HIV care	HIV/Sexual health	Young (16–34 years old) gay & bisexual men, & TG women living with HIV	Black (79%) Latino 13%, White 1%	91	16–34	*N/A*	*N/A*	*N/A*	Messaging Social media e-coaching	Targeted, tailored & personalised mHealth interventions can significant reduction in missed HIV care appointment, increase in viral load suppression among the participants.	[76, 77]
2016–2017	Quasi-experimental	SMARTtest	USA	To facilitate the use of the INSTIMultiplex for self & partner HIV & Syphilis testing	HIV/Sexual health	MSM & TG women	Non-Hispanic White (31%) Non-Hispanic African American (41%), Hispanic, Latinx (18%), Others (10%)	60	20–73	2	*N/A*	*N/A*	Smartphone application	The app can help facilitating correct use of HIV self-test, correct reading of results & linkage of care. Participants would like to save & share the results (especially non-reactive results)	[78]
2015–2017	Quasi-experimental	*N/A*	Thailand	Promote HIV pre & post-test counselling	HIV/Sexual health	MSM & TG women	Thai (100%)	571	18+	54 (intervention) 45 (control)	Mean age (27)	*N/A*	Videoconferencing	Online supervised HIV self-testing is highly acceptable by first-time HIV testers. TG people are more likely to choose online counselling & self-testing.	[79]
2015–2016	RCT	PrEPmate	USA	To enhance PrEP adherence	HIV/Sexual health	Young MSM	Latino (36%) Black (27%) White (25%) Asian (7%)	81 (intervention) 40 (control)	18–29	3 (intervention) 3 (control)	*N/A*	*N/A*	Messaging	Bidirectional text-messaging PrEP support is an acceptable, effective way to increase PrEP retention & adherence.	[42]
2013–2016	RCT	iTAB	USA	To improve PrEP adherence	HIV/Sexual health	MSM & TG women	White (74%) Black (13%) Asian (3%) Hispanic (30%)	200 (intervention) 198 (control)	19–64	3 (intervention) 0 (control)	*N/A*	*N/A*	Messaging	Text messaging may help maintaining drug adherence, although the results was not significant	[80]
2013–2015	RCT	CARE+ Corrections & CARE+ SMS	USA	To support ART adherence & linkage /engagement in community HIV care	HIV/Sexual health	Recently incarcerated persons with HIV	Non-Hispanic black (85%) Hispanic/others (11%) Non-Hispanic White (4%)	57 (intervention) 55 (control)	30–49	10 (intervention) 10 (control)	*N/A*	*N/A*	Messaging Learning platform	No statistically significant change was found in viral load & engagement in care between intervention & control arms.	[81]
2013	Qualitative evaluation	Virtually Trans	Australia	To support group members wellbeing & facilitate information exchange	Behavioural/psychosocial wellbeing	TG men & other TG people assigned female at birth	"Majority identified as white"	*N/A*	*N/A*	*N/A*	*N/A*	*N/A*	Social media	A secret Facebook page provides a safer platform for TG people to connect with each other.	[82]
2013	Retrospective data review	Gay City Wellness Center	USA	Prescribing PrEP	HIV/Sexual health	MSM & TG persons visiting a pilot PrEP clinic	White (38%) Hispanic (29%) Asian (8%) Black (6%)	10 (telehealth) 48 (usual care)	19–46	0 (telehealth) 1 (usual care)	*N/A*	*N/A*	Videoconferencing	Combining a specialist telehealth consultation & in-person PrEP counsellors who provide on-site blood collection & PrEP counselling appears to be a feasible, acceptable, & cost-effective approach to prescribe PrEP.	[83]
2012–2013	Quasi-experimental	HealthMpowerment	USA	To reduce risky sexual behaviours, promote health & wellness, & support community-building	HIV/Sexual health	Black MSM, & TG women	Black (100%)	15	20–30	5	*N/A*	*N/A*	Learning platform	The intervention was a feasible & acceptable tool to bring positive impact to participants’ health. Involving target population to help co-designing a e-health program is important	[43, 84, 85]
2010–2012	RCT	CARE+ Spanish	USA	To increase ARV adherence, reduce HIV viral load & sexual risk behaviours	HIV/Sexual health	Spanish speaking, HIV-positive cisgender & TG men & women	Latino (97%)	226 (intervention) 207 (control)	18+	9 (intervention) 1 (control)	*N/A*	*N/A*	Learning platform	No statistically significant change was found in ARV adherence, viral load & sexual transmission risk behaviours. However, the program was viewed positive, acceptable & linguistically appropriate by participants without requiring additional staff time.	[86]
*N/A*	Qualitative evaluation	U-Signal	USA	To provide support to the users’ safety, by sending help messages & GPS coordinates to a trusted friend in emergency	Behavioural/psychosocial wellbeing	TG & non-binary people of color	English speaking "trans persons of color"	16	*N/A*	16	*N/A*	Yes (16 trans & non-binary persons)	Smartphone application	The pilot intervention was perceived as highly acceptable because it utilised peer support networks.	[87]

Only 13 (33%) projects reported the age of transgender people recruited and 5 projects included transgender people under 18. The youngest and oldest participants recruited were documented as being 12 and 60+ years old, both being transgender patients of a health clinic [44]. Participants’ ethnic backgrounds followed the ethnic demographics of North America, with most projects having recruited participants who were reported as black/Latino/Hispanic or white.

### Overview of ICT interventions involved

Among the 39 ICT-based projects, a total of 8 major types of ICT interventions were described. These interventions included videoconferencing, smartphone applications, text-messaging, social media, telephone calls, e-coaching, self-learning platforms and e-consultation platforms. It is important to note that some of these interventions overlapped with each other and some projects used more than one ICT intervention.

#### Videoconferencing

Thirteen projects involved the use of videoconferencing technologies. Using platforms such as *Zoom*, *Google Meet* and *Doximity*, clinicians had conducted synchronous video-chat or audio-only consultations to provide gender-affirming and other care such as HIV and sexual health care [32, 48, 54], PrEP prescription [48, 83], pre-and post- HIV test counselling [66–68, 79], and behavioural counselling and support [46, 58]. For example, the *Open Door Health* LGBTQ+ clinic in Rhode Island used Zoom for patient encounters during the pandemic [48]. In other community settings without in-house physicians, such as at the Gay City Wellness Centre in Seattle, PrEP consultations were conducted virtually with an off-site physician using a computer in the centre, and venepunctures were done by on-site staff members [83]. All of the available literature points to the fact that videoconferencing is a highly acceptable, satisfactory and feasible approach to promoting service accessibility among geographically dispersed communities, especially during the pandemic.

#### Smartphone applications

In eleven projects smartphone applications were used, mostly to promote HIV and sexual health through increasing PrEP awareness [52, 72], providing HIV- and STI-specific information [47, 52, 53, 69], and improving linkage to HIV and STI services [50, 69, 72, 78]. Four projects specified that transgender people were involved in the development stage of these applications [47, 52, 53, 57]. Overall, smartphone applications have been reported in the literature as being effective and acceptable in improving HIV- and STI-related outcomes. Such applications typically consist of an education component that displays health information, and an activity component that includes games and self-risk assessments. Some applications, such as *TechStep* [57], *HealthMpowerment 2*.*0* [47] and *Trans Women Connect* [52], also contained interactive functions such as polling and forums that allowed users to connect. Others, such as *RUMAH SELA* [69] and *PrEPme* [72], involved a messaging function for users to connect with a peer worker or health educator to ask HIV-related questions or to arrange PrEP appointments. Several incorporated smartphones’ built-in capacities. For instance, *SMARTtest* [78] used smartphones’ cameras to help MSM and transgender women to interpret at-home HIV and syphilis test results. Users were then able to save and share the test results with doctors. Another app, *Geopassport* [50], assisted MSM and transgender women who had recently left prison to navigate HIV and related services. The application provided geolocation-based information and cash incentives when a person visited a service.

#### Messaging

Nine projects involved sending short messages through a telephone or computer, such as SMS, emails, or other instant messengers. Messaging was another common strategy to promote HIV prevention and care. Messaging in some cases was unidirectional and in other cases bidirectional, and participants were able to customise the types of messages they would receive and select the frequency and timing of messaging. For example, the interventions *iTab* [80], *Text Me*, *Girl*! [74], *TechStep* [57] and *CARE+ SMS* [81] sent pre-developed daily motivational text messages to participants in order to encourage adherence to PrEP or HIV treatment, and to promote emotional health. Some interventions, such as the *PrEPmate* study [42], utilised a bidirectional automated response system which required participants to respond to ‘check in’ messages asking about their PrEP use. Study staff then reached out to participants who indicated they needed further assistance or who did not respond to the messages within a set timeframe. Others, such as the *weCare* [76, 77] and *HealtheNav* [73] projects combined text messaging and one-on-one coaching. Participants in these projects were able to communicate with a designated coach or health educator through text messaging. Overall, the literature indicates that text messaging, especially bi-directional messaging, is an acceptable, effective and personalised way to improve medication adherence and other HIV-related health outcomes [42, 76, 77]. Moreover, bi-directional messaging also facilitates the collection of real-time data from participants and the provision of timely support [73].

#### e-Coaching

e-Coaching was mentioned in seven projects. In these projects, coaches (sometimes referred to as ‘mentors’ [50], ‘cyberhealth educators’ [76, 77] or ‘care navigators’ [73]) who were trained peer workers, paraprofessionals or professionals communicated with participants regularly. With the exception of one project in which coaches only met with participants in person [50], coaches in all other projects identified and utilised different ICT tools, e.g., text messaging [57, 62, 73, 76, 77], video conferencing [58], phone calls [62], emails [88], apps [76, 77] and social media [76, 77, 88] to connect with participants. In most projects, coaches were a rather casual point of contact who delivered personalised support to participants according to individual project aims. In the *SMART* project [58], however, coaches delivered more structural one-on-one counselling sessions to high-risk participants to encourage safer sex behaviors.

#### Self-learning platforms

Six different types of self-learning platforms were described in the literature. In general, these platforms consisted of education components which involved text, pictures and videos, and interactive components such as games, quizzes and forums. Some interventions, such as *Singularities*, incorporated elements of role-playing games and teenage players were able to learn about skills for preventing victimisation and bullying when completing various ‘missions’ in the game [59, 60]. Nevertheless, the effects of self-learning platforms alone on positive health change appeared to be small or negligible. While studies like *Singularities* demonstrated some improvements in certain short-term outcomes (e.g., help-seeking and knowledge of online resources) in the intervention group, the study was not powered to find significant effects for most secondary and tertiary outcomes [60]. Other platforms, such as *HealthMpower*, successfully engaged with young, black cisgender and transgender people through a points-based rewards system which allowed participants to exchange points they earned from doing online quizzes and games with real prizes [43, 84, 85], but there was only a marginally significant effect on change in safe-sex norms [85]. Similarly, the offline, HIV-clinic-based *CARE+Spanish* did not show any statistically significant effects on the medication adherence and viral loads of Spanish-speaking HIV-positive cisgender and transgender participants [86].

#### Telephone calls

Three projects described the use of telephone calls. Phone calls were used as an alternative strategy to connect with participants, such as when a transgender patient lacked access to the internet [54] or if they preferred to be contacted by phone over other strategies [62]. In some projects, telephone warmlines were run to engage with people who had questions with PrEP [72]. In the *Together 5000* study [63], positive test results of lab-based at-home HIV testing were delivered to cisgender and transgender participants over the phone by trained staff. The phone calls followed a standardised protocol that consisted of mental health assessment and provision of referrals to facilitate linkage to care. Based on evaluation with cisgender participants (no transgender persons were recruited in the evaluation), the authors found that in general at-home HIV testing with phone delivery of results could be an acceptable and feasible way to increase HIV testing access to geographically isolated people. Compared to email and online portals, phone calls were considered the more favourable means for staff to deliver emotional support to users preferring instant, live responses [63].

#### Social media

Three projects [62, 76, 77, 82] involved the use of invite-only ‘secret’ social media platforms to connect transgender people. These projects found that social media provided a confidential and safe platform for meaningful exchange of information between peers, and in turn promote health by filling social support, resource and knowledge gaps for participants, especially for those who were younger. Projects such as *weCare* combined social media with one-on-one coaching and significantly reduced missed medical appointments and increased viral load suppression among cisgender and transgender participants with HIV [77].

#### e-Consultation platforms

Two projects [64, 65] described the use of e-consultation platforms to link primary health providers with transgender health specialists. Using these platforms, clinicians who were not familiar with gender-affirming care were able to seek support from a wider network of specialists, thereby increasing the access and linkage of transgender people to appropriate care, especially for those who lived in rural areas. Moreover, analysing the questions that providers asked could also help researchers in identifying potential gaps in knowledge among primary care providers. However, some challenges included delayed response from specialists, and specialists may provide impractical recommendations because they did not understand the context and difficulties of under-resourced settings [64].

### Concerns about ICT-based trans healthcare delivery and ways to address them

Below we present five concerns on delivering ICT-based transgender healthcare and ways to address them that we identified from the literature, based on critical appraisals of the methodologies and discussions of the individual papers. It should be noted that as the majority of the articles we reviewed only consisted of a small number of transgender people, the suggestions on addressing concerns summarised below are, to some extent, general and may apply to other LGBTIQ+ populations.

#### Content appropriateness and relevance

Adopting a user-centred approach is an essential component in maintaining the appropriateness and relevance of an ICT intervention [43, 46, 76]. Many projects consulted community members in the early stages, such as by conducting focus groups [42, 80] or setting up advisory boards [47, 52, 53, 57, 62, 74, 89], to allow the target population to become an active part of the creative development of the intervention. Nevertheless, most of these projects did not specify the composition of the advisory boards. Only five projects indicated that the development of the intervention involved direct input from transgender people [47, 52, 53, 57, 74]. It has to be noted, though, that some of these projects only involve two [47] or three [52] transgender persons in the consultation process and it was unclear how their voices were adequately represented in the process.

#### Safety and confidentiality concerns

Confidentiality and safety of intervention recipients were concerns documented in the literature [32, 46, 48, 63, 73]. For example, some participants were concerned that unauthorised third parties could intercept or eavesdrop on technology-delivered health-related messages [46, 63, 86]. This was partly because some of this communication was done at an inopportune time of day such as early in the morning or when clients were at work [63]. Some possible ways to create a virtual environment where people feel safe and comfortable to share their personal issues included setting up privacy protection components and clear instructions for users, such as advising them to use headphones [86]. For some younger transgender persons, they may feel uncomfortable seeing themselves on the computer screen possibly due to dissatisfaction with their own bodies [32]. In order to create a safer and more welcoming virtual setting, healthcare providers should assess each service recipient’s comfort with the video function and allow them to disable the camera if needed [32]. Ongoing software updates and maintenance are also important [73] to keep ICT interventions secure, safe and relevant.

#### Limited literacy and access to technology

Several articles reported challenges in regard to recipients’ literacy level and access to technology [42, 46, 48, 86]. Some interventions enrolled only people with access to computers, smartphones and the internet [68]. Older people sometimes found it difficult to use technology-based services [46, 48, 53]. Poor and unstable internet connections led to glitches that disrupted access to services [46, 79, 90]. Appropriate assistance should be provided to help intervention recipients to overcome technical challenges, such as assisting disadvantaged people to address financial barriers related to the costs and access to equipment. Designing interventions using platforms familiar to the target populations (e.g., sending treatment information via social media platforms such as Facebook) was considered as another possible way to facilitate users to adopt a new intervention [76]. Multiple platforms should also be considered when attempting to reach transgender people of diverse backgrounds and needs [52].

#### Physical examinations and suboptimal interpersonal contacts

Suboptimal interpersonal contacts in virtual settings may hinder health providers’ ability to perform complete physical examinations and take sample collections [32, 83]. In one project, pathology tests were delayed for virtual consultations, and physical exams were replaced by visual assessments, patient self-exams and self-collected vital signs [48]. Getting parental consent for initiating gender-affirming hormones for transgender youth could also be challenging in an online setting [48]. Therefore, some younger transgender people may prefer in-person visits compared to videoconferencing for sexual health care, surgical consults, and initial gender care visits [32].

#### Infrastructural and legal support

The broader legal and health infrastructure affects the delivery of ICT-based transgender healthcare. A challenge described in some studies is the absence of adequate legal frameworks in some countries or localities that are supportive of remote care delivery [79, 83]. Cross-jurisdictional telehealth could be a problem when clinicians are limited to practicing in specific jurisdictions with their licence [44]. Moreover, studies conducted in the USA highlighted ongoing concerns among providers and clients in regard to the billing of virtual care [48, 76, 83]. In relation to this, the USA Health Insurance Portability and Accountability Act (HIPAA) regulations had to be relaxed first so that many providers could adopt diverse technologies (including those that are not HIPAA compliant) to care for their clients during the COVID 19 pandemic [90].

## Discussion

This scoping review is among the first to synthesise research on ICT-based interventions for transgender people. Our review has identified 39 ICT-based projects/programs, 8 types of interventions, and 5 common considerations for developing effective interventions for transgender people. Consistent with the broader literature [7, 8], the range of interventions identified demonstrate the huge potentials of ICT to improve healthcare access for transgender people in relation to its flexibility and convenience. Nevertheless, while the body of literature has been expanding rapidly in the past two years, transgender people have too often been regarded as a small sub-sample in ICT-based programs and research. In some studies, transgender people have only represented as low as around 0.1% of the total sample size [73, 78]. With such a small sample size, studies may not be adequately powered to detect the effectiveness of interventions for transgender people. Many studies did not specificize the involvement of transgender people in the design or evaluation process. Some studies [42, 43] even misclassified transgender people as men who have sex with men or transgender as a sexual identity. All these findings point to a lack of programs and research that are transgender people specified.

Three major gaps were noted in the literature. Firstly, relatively little is known about the implementation of ICT-based trans health interventions outside the context of HIV and sexual health. Although it has been widely argued that ICT can be adopted in other fields, such as mental health care, gender-affirming care and perioperative care for transgender people [8, 35], only three studies discussed how ICT-based gender-affirming care could be provided in real-world clinical settings. This finding is consistent with a recent review on mobile-based interventions targeting transgender youth that most interventions have been focusing on HIV and sexual health care [37]. The three articles indicated some practical considerations regarding camera use [32], specimen collection, and consent process [48] that warrant future studies.

Secondly, there is a lack of global evidence, and how ICT-based transgender healthcare can be best used in resource-limited settings is yet unanswered. While this review included studies from Asia and Australia, most of the included articles were conducted in the USA. Implementing ICT-based interventions in resource-limited settings have both challenges and possibilities. On the one hand, there are barriers in relation to technology access, people’s computer literacy, financial support, and regulation of telemedicine [91]. Poverty, human rights situations and socio-political contexts also likely affect transgender people’s access to and experience of ICT-based interventions [92]. On the other hand, opportunities exist as healthcare is often provided directly by community peer workers or paraprofessionals in resource-limited settings, a tele-supervision model that links frontline workers to remote specialists may help improve access substantially [93]. This review included similar interventions in the USA that involved linking primary health providers with transgender health specialists, and interventions that supplemented frontline healthcare by community workers with teleconsultations with off-site physicians [83]. Future research can explore the most effective models of ICT-based interventions for transgender people living in different socioeconomic settings.

Thirdly, more data are needed to understand the access and experience of subpopulations of transgender people. The transgender population is highly heterogeneous and consists of differing races/ethnicities, ages and other intersectional characteristics. As the current literature is dominated by studies from the USA, the racial/ethnic profiles of participants in these studies resemble the demographics of the country which are dominated by white, black and Hispanic people. Interventions have often targeted transgender people who are younger (usually under 30 years), English or Spanish speaking, and who were assigned male at birth. Future studies could explore how to enhance intervention inclusiveness across diverse ethnic and language groups, such as Asian, Pacific Islander and Indigenous peoples, those assigned female at birth, and those who are older.

Although the current body of evidence is still far from adequate for us to make comprehensive recommendations on the best practice of ICT-based interventions for transgender people, the small body of literature has still provided some insights into effective interventions. Co-developing interventions with target participants to improve the interventions’ appropriateness is a case in point. Nevertheless, researchers and health providers must be aware of the risk of tokenism and be transparent in how transgender people are engaged in the consultation, development and implementation process. In our review, only a small number of articles have specified that transgender people were involved directly in the development of the interventions. Another pertinent point to consider is maintaining humanistic interactions in ICT-based interactions. For example, compared to email and online portals, phone contacts were considered the more favourable means for staff to deliver emotional support to users who prefer instant response and a real person to listen [63]. In addition to professionally trained practitioners such as doctors and nurses, peer workers with lived experience of similar issues add significant value to ICT-based interventions, especially for interventions that target the community of colour [72]. More importantly, the success of an intervention depends not only on the intervention itself, but also on the broader socio-political context such as how transgender people are supported by the legal and health systems. Systematic adjustments and health advocacy, such as, establishing a payment policy for telemedicine and allowing longer supplies of medication [48], changing one’s gender marker or name on identity documents [82, 94], and adjusting licensure requirements for cross-border telehealth programs [95], are necessary for ICT-based interventions to scale up and better link with other components of the healthcare system.

### Limitations

Similar to most scoping reviews, while our use of comprehensive search terms and the practice of citation mining allowed us to capture a wide range of relevant publications, it was not possible to identify all literatures in the field. It is important to note that the ways and terms by which transgender people identify themselves are rooted in specific sociocultural contexts [96], making it difficult to capture all transgender people. Our search strategy also limited the review to literatures that are accessed through three databases, and the databases we used had their strengths and limitations (e.g., the use of MeSH terms did not cover ahead-of-print articles that have not been indexed, and Google scholars tended to pick up irrelevant articles). The search potentially favored articles published from resource-privileged and English-speaking countries, although a trial search was also conducted in a Chinese-language database which yielded no relevant articles. Including only peer-reviewed articles in this scoping review was another limitation. The literature varied in designs, sample size, and rigour, and some of the articles reviewed were research protocols, and therefore the degree to which their results could be compared to each other, and the transferability of their conclusions was unclear. Despite these shortcomings, we believe that our efforts help summarise and identify knowledge gaps on the types of work that have been done on this topic, thereby informing further work in this area.

## Conclusions

While there is a rapidly growing literature on the use of information and communication technology to deliver healthcare to sexual minorities, transgender people are often still regarded as a sub-sample in most ICT-based programs and research. Future interventions, in addition to HIV and sexual health, should also focus on other unique health needs/issues that transgender people experience such as mental health and gender affirming care. It can also be of great importance to explore how ICT can be used to improve the health of transgender people from diverse sociocultural backgrounds. Crucially, the success of an ICT-based intervention depends not only on the intervention itself, but also on how transgender people are supported in the broader socio-political context. Health researchers have a responsibility to adopt a more inclusive approach in the design and delivery of interventions that are safe, accessible and effective, while continuing to advocate for the rights and health of transgender people from different policy perspectives.

## Supporting information

S1 Checklist PRISMA -ScR checklist. (DOCX)

S1 File Search strategies. (DOCX)
