# Supplementary material for: Information and communication technology-based health interventions for transgender people: A scoping review
Source: PLOS Glob Public Health. 2022 Sep 15;2(9):e0001054. doi: 10.1371/journal.pgph.0001054 (PMC10021903; doi:10.1371/journal.pgph.0001054)
Supplement: S1 File — (DOCX) [file pgph.0001054.s002.docx]

**S1 File: Search strategy**

Literature search was conducted using the following search terms. The search queries were first developed for PubMed using medical subject headings (MeSH), and then extended to two scientific databases: CINAHL and Scopus. Search terms in CINAHL and Scopus were modified to fit the databases’ functionalities. The first search was conducted in May 2021. In January 2022, a supplementary search was conducted to identify more recent articles published between May and December 2021.

**Database 1: Pubmed**

| Search | Query |
| --- | --- |
| #1 | **"Telemedicine"[Mesh] OR "Mobile application*"[tw] OR "mobile app"[tw] OR "mobile apps"[tw] OR "mobile technolog*"[tw] OR "information communication technolog*"[tw] OR ICT OR "mobile health"[tw] OR mHealth OR Telehealth[tw] OR eHealth[tw] OR Telemedicine[tw] OR "smartphone"[tw] OR "digital health"[tw] OR ecare[tw] OR mcare[tw] OR telecare[tw] OR “Cellular phone*”[tw] OR “digital technolog*”[tw] OR “telecommunication*”[tw] OR “information technolog*”[tw]** |
| #2 | "Delivery of Health Care"[Mesh] OR "Delivery of Health Care, Integrated"[Mesh] OR "Health Education"[Mesh] OR "health education"[tw] OR "health promotion"[tw] OR "delivery of healthcare"[tw] OR "consultation"[tw] OR "Health care"[tw] OR healthcare[tw] OR "health service"[tw] OR "health provision" OR "Delivery of Health services"[tw] OR "delivery of healthcare" OR “Health Services Accessibility”[Mesh] OR “Health Services Accessibility” |
| #3 | **"****Transgender Persons"[Mesh] OR "Health Services for Transgender Persons"[Mesh] OR "Sexual and Gender Minorities"[Mesh] OR "Gender diverse"[tw] OR "non-binary"[tw] OR "transgender"[tw] OR "sexual minorit*"[tw] OR "gender minorit*"[tw] OR "transman"[tw] OR "transwoman”[tw] OR Transmasculine [tw] OR Transfeminine[tw] OR genderqueer[tw] OR "gender fluid"[tw] OR "Gender non-conforming" OR "Cross sexual"[tw] OR "transsexual"[tw]** OR “Gender Equity”[mesh] OR “gender equity”[tw] OR “Gender Affirmation Procedure*”[tw] OR “Gender identity”[mesh] OR “gender identity”[tw] OR “Gender Specific Care”[tw] |
| #4 | Search: #1 AND #2 AND #3 |

**Database 2: CINAHL**

| Search | Query |
| --- | --- |
| #1 | (MH "Telehealth+") OR (MH "Cellular Phone+") OR (MH "Digital Technology+") OR (MH "Telecommunications+") OR (MH "Information Technology+") OR “telemedicine” OR “mobile application*” OR “mobile app” OR “mobile apps” OR “mobile technolog*” OR “information communication technolog*” Or “ICT” OR “mobile health” OR “mhealth” OR “telehealth” OR “ehealth” OR “telemedicine” OR “smartphone” OR “digital health” OR “ecare” OR “mcare” OR “telecare” |
| #2 | (MH "Health Care Delivery, Integrated") OR (MH "Health Care Delivery+") OR (MH "Health Services Accessibility+") OR (MH "Health Education") OR (MH “Health promotion”) OR "Delivery of health care” OR “Delivery of health care, integrated” OR “Health education” OR “consultation” OR “Health care” OR “healthcare” OR “health service” OR “health provision” OR “delivery of health services” OR “delivery of healthcare” |
| #3 | MH "Sexual and Gender Minorities") OR (MH "Gender Affirmation Procedures") OR (MH "Gender Nonconformity") OR (MH "Sexual and Gender Disorders") OR (MH "Gender Specific Care") OR (MH "Gender Equality") OR (MH "Gender Identity") OR (“transgender persons” OR “Health services for transgender persons” OR “sexual and gender minorities” OR “gender diverse” OR “non-binary” OR “transgender” OR “sexual minorit*” OR “gender minorit*” Or “transman” OR “Transwoman” OR “transmasculine” OR “transfeminine” OR “genderqueer” OR “gender fluid” OR “gender non-conforming” OR “cross sexual OR transsexual” |
| #4 | Search: #1 AND #2 AND #3 |

**Database 3: Scopus**

| Search | Search Terms |
| --- | --- |
| #1 | ( ALL ( “telemedicine”  OR  “mobile app”  OR  “mobile application”  OR  “mobile tech*”  OR  “information communication”  OR  “mhealth”  OR  “telehealth”  OR  “ehealth”  OR  “smartphone”  OR  “digital health”  OR  “ecare”  OR  “mcare”  OR  “telecare”  OR  “Cellular phone”  OR  “cellphone”  OR  “digital tech*” )  AND  ALL ( “health service delivery”  OR  “delivery of health care”  OR  “Health education”  OR  “health promotion”  OR  “consultation”  OR  “healthcare”  OR  “Health care”  OR  “health provision”  OR  “health services accessibility” )  AND  ALL ( “Transgender”  OR  “sexual and gender minorit*”  OR  “transsexual”  OR  “SGM”  OR  “gender divers*”  OR  “non-binary”  OR  “transman”  OR  “transwoman”  OR  “Transmasculine”  OR  “Transfeminine”  OR  “genderqueer”  OR  “Genderfluid”  OR  “Gender non-conforming”  OR  “cross sexual”  OR  “gender equity”  OR  “gender equality”  OR  “gender affirmation”  OR  “gender specific care” ) ) |

**Additional literature explorations using Google Scholar and the Chinese language database, Airiti Library**

**Google scholar:**

| **"Telemedicine" OR "Mobile application" OR "mobile app" OR "mobile apps" OR "mobile technology" OR "information communication technology" OR "ICT" OR "mobile health" OR "mHealth" OR "Telehealth" OR "eHealth" OR "Telemedicine" OR "smartphone" OR "digital health” AND "Delivery of Health Care" OR "Delivery of Health Care” OR "Health Education" OR "health education" OR "health promotion" OR "delivery of healthcare" OR "consultation" OR "Health care" OR “healthcare” OR "health service" OR "health provision" OR "Delivery of Health services" OR "delivery of healthcare" OR “Health Services Accessibility” OR “Health Services Accessibility” AND "Transgender Persons" OR "Health Services for Transgender Persons" OR "Sexual and Gender Minorities" OR "Gender diverse" OR "non-binary" OR "transgender" OR "sexual minority" OR "gender minority" OR "transman" OR "transwoman” OR “Transmasculine” OR “Transfeminine” OR “genderqueer” OR "gender fluid" OR "Gender non-conforming" OR "Cross sexual" OR "transsexual" OR “Gender Equity” OR “gender equity” OR “Gender Affirmation Procedure” OR “Gender identity” OR “gender identity” OR “Gender Specific Care”** |
| --- |

**Notes:**

- Only selected search terms were used because Google scholar searched full text not specific fields like title, abstract, keywords, subject headings, etc. There it retrieved more results with less relevancy.

**Airiti Library**

| ([DC]:(telemedicine) OR [ALL]: (遠距) OR [ALL]: (app) OR [ALL]:(technolog) OR [ALL]:(ICT) OR [ALL]:(mobile) OR [ALL]:(mhealht) OR [ALL]:(telehealth) OR [ALL]:(phone) OR [ALL]:(digital) OR [ALL]:(ecare) OR [ALL]:(mcare) OR [ALL]:(telecare) OR [ALL]:(telecommunication)) AND [ALL]:(delivery) OR [ALL]: (health care) OR [ALL]: (醫療) OR [ALL]: (education) OR [ALL]:(promotion) OR [ALL]:(consultation) OR [ALL]:(accessibility)) AND [ALL]:(transgender) OR [ALL]:(sexual) OR [ALL]:(gender) OR [ALL]:(nonbinary) OR [ALL]:(trans) OR [ALL]:(queer) OR [ALL]:(nonconforming) OR [ALL]:(cross sexual) OR [ALL]:(transsexual) OR [ALL]:(gender equity) OR [ALL]:(gender identity) OR [ALL]:(gender specific care) OR [ALL]: (跨性別)) |
| --- |

**Notes:**

- Airiti Library is one of the largest Chinese-language academic databases that features academic resources of both Taiwan and Mainland China. Search results included all Traditional and Simplified Chinese journal articles, conference proceedings, theses, eBooks and Taiwanese’s National Palace Museum (NPM) Periodicals.
- Only selected search terms were used because the platform does not allow complex searching with search terms more than 400 characters.
- The trial search retrieved zero results and was not included in the final search strategy.
